# MetaTrass: A high‐quality metagenome assembler of the human gut microbiome by cobarcoding sequencing reads

**DOI:** 10.1002/imt2.46

**Published:** 2022-08-15

**Authors:** Yanwei Qi, Shengqiang Gu, Yue Zhang, Lidong Guo, Mengyang Xu, Xiaofang Cheng, Ou Wang, Ying Sun, Jianwei Chen, Xiaodong Fang, Xin Liu, Li Deng, Guangyi Fan

**Affiliations:** ^1^ BGI‐Qingdao BGI‐Shenzhen Qingdao China; ^2^ State Key Laboratory of Agricultural Genomics BGI‐Shenzhen Shenzhen China; ^3^ China National GeneBank BGI‐Shenzhen Shenzhen China; ^4^ College of Life Sciences University of Chinese Academy of Sciences Beijing China; ^5^ BGI‐Shenzhen BGI‐Shenzhen Shenzhen China; ^6^ MGI BGI‐Shenzhen Shenzhen China; ^7^ BGI Genomics BGI‐Shenzhen Shenzhen China

**Keywords:** metagenome assembly, microbiome composition, synthetic long reads, taxonomic binning

## Abstract

Metagenomic evidence of great genetic diversity within the nonconserved regions of the human gut microbial genomes appeals for new methods to elucidate the species‐level variability at high resolution. However, current approaches cannot satisfy this methodologically challenge. In this study, we proposed an efficient binning‐first‐and‐assembly‐later strategy, named MetaTrass, to recover high‐quality species‐resolved genomes based on public reference genomes and the single‐tube long fragment read (stLFR) technology, which enables cobarcoding. MetaTrass can generate genomes with longer contiguity, higher completeness, and lower contamination than those produced by conventional assembly‐first‐and‐binning‐later strategies. From a simulation study on a mock microbial community, MetaTrass showed the potential to improve the contiguity of assembly from kb to Mb without accuracy loss, as compared to other methods based on the next‐generation sequencing technology. From four human fecal samples, MetaTrass successfully retrieved 178 high‐quality genomes, whereas only 58 ones were provided by the optimal performance of other conventional strategies. Most importantly, these high‐quality genomes confirmed the high level of genetic diversity among different samples and unveiled much more. MetaTrass was designed to work with metagenomic reads sequenced by stLFR technology, but is also applicable to other types of cobarcoding libraries. With the high capability of assembling high‐quality genomes of metagenomic data sets, MetaTrass seeks to facilitate the study of spatial characters and dynamics of complex microbial communities at enhanced resolution. The open‐source code of MetaTrass is available at https://github.com/BGI-Qingdao/MetaTrass.

## Highlights

- MetaTrass is the first metagenome assembly tool implemented by the binning‐first‐assembly‐later strategy and it is freely available at https://github.com/BGI-Qingdao/MetaTrass.
- MetaTrass integrates the cobarcoding and the reference genome information to achieve a high capability of generating high‐quality genomes at the species level for the human gut microbial communities.
- MetaTrass uses the cobarcoding correlation between short‐length reads to reduce false‐negatives in conventional taxonomic binning and improve the contiguity of draft assemblies.

## Graphical abstract

We developed a tool to get high‐quality genomes with high taxonomic resolution by combining the cobarcoding information with public references. Compared with the conventional combination strategies, our pipeline generated a large number of high‐quality genomes for the human microbial cobarcoding data sets.

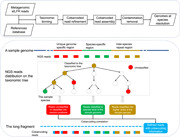

## INTRODUCTION

Through sequencing and analyzing the DNA of microbial communities directly from the environment, metagenomics has shown significant promise in advancing the study of uncultured microbiomes [1, 2]. A high level of genetic diversity has been unveiled by the growing number of comprehensive metagenome‐assembled genomes (MAGs), particularly from human gut microbiomes [3, 4]. The progress in metagenomics has shed new light on the study of spatial distribution and dynamics of complex microbial communities from the human gut [5–7].

Functional mining of high‐quality strain‐resolved genomes revealed a strong correlation between genotypic differences among strains and their phenotypic differences [8, 9]. Intra‐species nonhomologous genes can serve as biomarkers to distinguish pathogenic strains from their commensal counterparts within a species [10–12]. The percentage of conserved intra‐species homologous genes shared among strains could be as low as 40% [13], and the remaining nonconserved genome sequences are considered to have a significant contribution to the phenotypic diversity of microorganisms. Therefore, complete genomes from a microbial sample at the species level will enable a more comprehensive view of intra‐species genome diversity. But it is still a challenge to generate sufficient high‐quality genomes from metagenomic data sets.

Most of the current approaches to analyzing microbial communities are designed to work with economical next‐generation sequencing (NGS or high‐throughput sequencing [HTS]) reads [14]. Many highly modularized computational tools have been developed, including genome assemblers, genome binners, taxonomic binners, and taxonomic profilers [15–17]. The assembly‐first‐and‐binning‐later strategies have been commonly used to generate MAGs. In these conventional strategies, short reads from a microbial community are first assembled into longer sequences by metagenomic assemblers with the consideration of uneven coverage depths of different microbial species [18–20] and further grouped into individual genomes by genome binners based on *K‐mer* composition and read coverage [21–23]. However, these conventional strategies often failed to resolve the long inter‐species repeats during contig reconstruction. Therefore, the contiguity of the draft genomes recovered from NGS reads remains not long enough to study large structural variations in microbial genomes.

Various sequencing technologies with long‐range information accompanied by specialized computational tools have been released to overcome the problem of long repeats. The third‐generation single‐molecule real‐time sequencing (TGS) technologies developed by Pacific Biosciences and Oxford Nanopore Technology (ONT) can produce contiguous reads with lengths up to hundreds of kilobases, and they show great potential to generate complete genomes from both cultured and uncultured microbial communities [24–26]. With the use of chromatin‐level contact probability information generated by high‐throughput chromosome conformation capture (Hi‐C) technology, more high‐quality MAGs with improved contiguity can be retrieved [27]. For NGS data sets, the coabundance of species in multiple samples with the common *K‐mer* composition is also used to improve the capability to achieve better assembly quality [28]. However, these approaches have several limitations. The high sequencing error rate in TGS long reads hampers the distinction between true biological variations and sequencing errors. An effective contact map with an Hi‐C library can only be established for a draft genome with long contiguity. Constructing coabundance in multiple samples ignores the genome characteristics of a single sample and increases the sequencing cost.

The cobarcoding sequencing library [29–33], an improved short‐read sequencing technology with long‐range genomic information, can provide an alternative way to improve metagenomic analysis. In cobarcoding library construction, long fragments of DNA molecules are first distributed into different isolated partitions and further sheared into shorter subfragments. Then these subfragments are indexed with a unique barcode (DNA cobarcoding). Finally, the cobarcoded subfragments are sequenced by standard short‐read sequencing platforms. For different cobarcoding libraries, such as BGI's single tube long fragment reads (stLFR) [32], 10X Genomics' linked‐reads [34], and Illumina's contiguity preserving transposase sequencing [30], technical differences in the number of barcodes and the short‐read coverage of the original long fragment have a great impact on the downstream analysis [35–38]. The cobarcoding correlation on the draft sequences or the assembled graph has been successfully applied to improve the contiguity of assembled genomes for both large eukaryotic genomes [39–41] and metagenomes [31, 42, 43]. However, none of these conventional strategies can conquer the inherent problem of long repeats among species with uneven abundance for complex microbial communities.

In this study, we introduce a pipeline named *Meta*genomic *T*axonomy *R*ead *A*ssembly of *S*ingle *S*pecies (MetaTrass) based on cobarcoding sequencing data and reference genomes. Unlike conventional strategies, MetaTrass is featured with an integrative binning‐first‐and‐assembly‐later approach. The cobarcoding information is mainly used for two purposes. First, it facilitates the initial taxonomic binning procedure by clustering reads with the same barcode. Second, it improves the assemblies by providing long‐range information. We applied MetaTrass to stLFR data sets of a mock microbial community and four human gut microbial communities to evaluate its capability of producing high‐quality draft genomes with long contiguity and high taxonomic resolution. The microbiome composition and genetic diversity in the four human gut microbial communities were quantitatively analyzed using the high‐quality draft genomes assembled by MetaTrass. In addition, the high‐quality draft genomes assembled by MetaTrass have clear taxonomic information determined by the taxonomic binning, thus benefiting the microbial downstream analysis. All results were benchmarked by comparison with the existing mainstream tools.

## RESULTS

### MetaTrass pipeline

**Figure 1:**
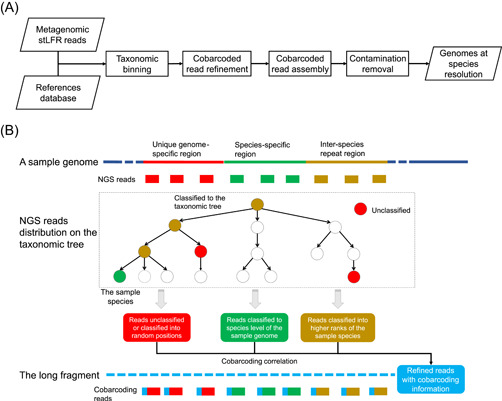
Flowchart and scheme of MetaTrass. (A) Flowchart of the MetaTrass assembling pipeline. (B) Scheme of cobarcoding correlation and taxonomic distribution of reads from different regions.

In this study, we developed a metagenome assembly pipeline named MetaTrass that integrates the information of reference genomes and DNA cobarcoding technology. As shown in the flowchart (Figure 1A), the taxonomic binning of stLFR reads was carried out before the genome assembling, unlike conventional assembly‐first‐and‐binning‐later strategies. In taxonomic binning, the metagenomic stLFR reads were classified into different taxonomic ranks by Kraken2 [44]. Due to the inherent limitations of Kraken2, only reads from conserved species‐specific regions can be successfully classified into the corresponding species. On the contrary, reads from inter‐species repeat and unique genome‐specific regions could not be effectively classified (Figure 1B). Here, the species‐specific regions represent similar or repeat sequences among genomes from the same species. The inter‐species repeat regions represent similar or repeat sequences among genomes from different species. The unique genome‐specific regions represent dissimilar sequences between genomes from the same species. The reads from inter‐species repeat regions are classified into the higher taxonomic ranks of the target species and those from unique genome‐specific regions are categorized as unclassified. In total, about 10% of the reads were classified into ranks higher than the bacterial species level for the four human fecal data sets and about 9% of the reads were unclassified (Supporting Information: Table S1). In the step of cobarcoded read refinement, the cobarcoding correlation between the reads from species‐specific regions and those from inter‐species repeat or unique genome‐specific regions was used to refine the final read set for a target species (Supporting Information: Figure 1B). The barcodes of the species‐specific reads were first extracted as the candidate barcodes. Then, the final barcodes were collected by a constraint of data size and the quality of cobarcoding information. Finally, reads of the target species were collected based on the final barcodes to be assembled in the following step. A data size threshold was set to reduce computational consumption for the species with extremely high abundance. The data size threshold was set to 300× by default according to the parameter sweep results of the sample P_Gut_Meta01 (Supporting Information: Table S2). The quality of cobarcoding information of a specific barcode was quantified by the number of reads with a confident species classification and the proportion of these reads among the total reads. In cobarcoded read assembly, the refined reads of each species were independently assembled by Supernova [39]. As aforementioned, there is a very small chance that long fragments from different species could be indexed with the same barcode in the stLFR libraries (Supporting Information: Figure S1). So, some false‐positive reads were introduced in the cobarcoded refinement procedure, and they were assembled to form contaminant sequences in draft assemblies. In the step of contamination removal, we eliminated these contaminant assemblies according to their dissimilarity to the reference genomes. The thresholds of average nucleotide identity (ANI) and alignment fraction (AF) were set to 90% and 50% by default according to the optimal results of the sample P_Meta_Gut01 (Supporting Information: Table S3). Overall, the comprehensive use of cobarcoding information and references in our approach could reduce the false‐negative effects of taxonomic binning and the false‐positive effects of cobarcoded read refinement.

### Assembly of the mock microbiome

**Figure 2:**
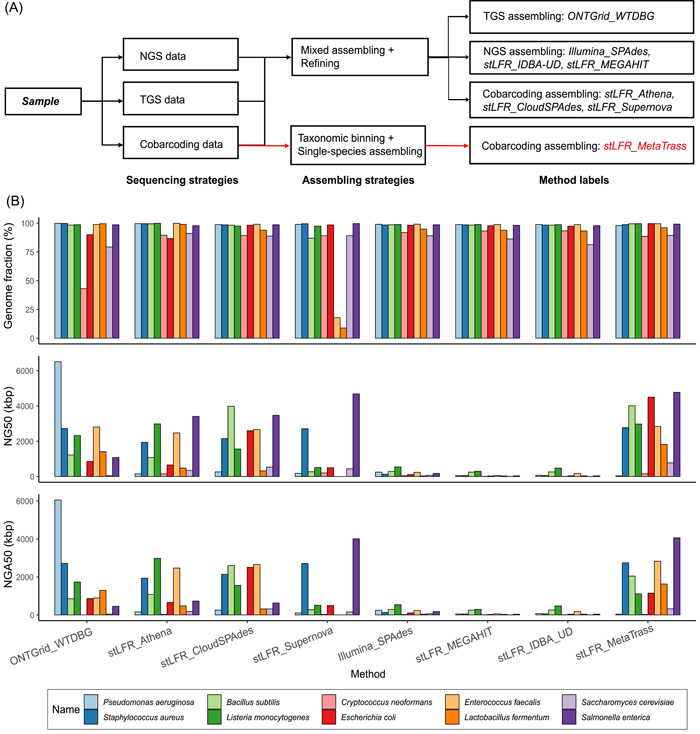
Scheme and evaluations for different strategies. (A) Difference labels of the assemblies based on different sequencing and assembling strategies. (B) Genome fraction, NG50, and NGA50 evaluated by QUAST for the assemblies.

The binning‐first‐and‐assembly‐later strategy has been widely adopted to assemble haplotype genomes for eukaryotes with large genome sizes [36, 45]. However, it has rarely been used to assemble metagenomes. We first applied MetaTrass to assemble stLFR read sets of the mock microbial community. In total, up to 99.4% of the reads were confidently assigned species‐level taxonomy given the simplicity of the mock microbial community with low inter‐species repeats and unique genome‐specific regions (Supporting Information: Table S4). To investigate the efficiency of our strategy, we compared it with the mainstream mixed assembly approaches (Figure 2A). Besides the MetaTrass analysis, the stLFR reads were also directly assembled by IDBA‐UD [20], MEGAHIT [18], Supernova [39], CloudSPAdes [42], and Athena [31]. Additionally, the optimal mixed assemblies of ONT and Illumina NGS reads in Nicholls's work [46] were also included for comparison, and assembled by WTDBG [47] and by SPAdes [48], respectively. The draft genome of each species in a mixed assembly was refined by our contamination removal module.

Overall, our pipeline was superior in the production of draft genomes with high genome fractions and long contiguity (Figure 2). Two species, *Enterococcus faecalis* and *Lactobacillus fermentum*, were incompletely assembled by Supernova with genome fractions as low as 17.7% and 8.9%. On the contrary, both species were properly recovered by MetaTrass, indicating that the assembly complexity caused by uneven abundances was reduced by taxonomic binning. When compared to the results of TGS reads assembled by WTDBG, the assemblies by MetaTrass had high genome fractions, similar to those by NGS or cobarcoding assemblers designed for metagenomes. As compared with the NGS assemblers, the cobarcoding and TGS assemblers produced draft genomes with better contiguity, among which MetaTrass showed the best performance. MetaTrass produced seven draft assemblies with NG50 around 2 Mb and obtained the highest number of assemblies with NGA50 around 2 Mb. Further, MetaTrass yielded fewer assembly errors as compared to the TGS assembler (Supporting Information: Figure S2). The average numbers of mismatches and indels per 100 kb in assemblies of stLFR reads were 60 and 10, respectively, which were smaller than those of the ONT assemblies. It was worth noting that the improvement of the assembly of *Pseudomonas aeruginosa* genome by MetaTrass was not large, and this may come from the fact that this species had the least amount of valid cobarcoding information (Supporting Information: Table S5). The valid cobarcoding information about each species was evaluated by counting the number of valid long fragments from each species. A valid fragment was defined as a cluster with more than five paired‐end reads and a maximum distance between two paired‐end reads longer than 10 kb, by aligning paired‐end reads to reference.

### Assembly of four human gut microbiomes

To evaluate the robustness of our approach, we applied MetaTrass to four human fecal samples to study human gut microbial communities. The comprehensive genome references of the Unified Human Gastrointestinal Genomes (UHGG V1.0) were used to classify NGS reads by Kraken2 [44], and the community compositions were estimated through the classified reads at different taxonomic ranks (Supporting Information: Figure S3–S6). The three healthy samples had a similar microbial community, in which the major microbiomes were from the bacterial phylum *Firmicutes A*. This microbial community was different from the patient microbial community dominated by the bacterial phylum *Proteobacteria*, which is demonstrated to be strongly correlated with enteric diseases caused by dysbiosis in gut microbiota [49]. The numbers of species with an abundance higher than 10× were 113, 108, 93, and 158 in samples H_Gut_Meta01, H_Gut_Meta02, H_Gut_Meta03, and P_Gut_Meta01, respectively.

**Figure 3:**
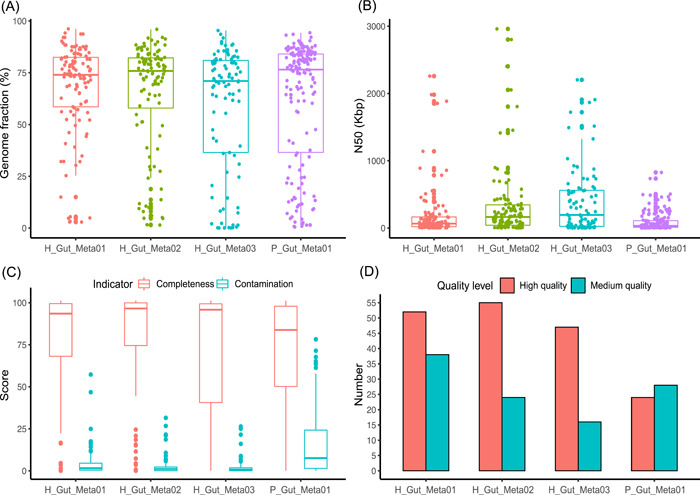
QUAST and CheckM evaluations of MetaTrass assemblies for the four human fecal samples. (A) Genome fraction. (B) Scaffold N50. (C) Box plot of completeness and contamination. (D) Number of high‐ and medium‐quality genomes

The genome fraction of an assembly to the reference was used to evaluate the completeness of genome assembly. The genome fraction for all samples widely ranges from 0% to 90%, and the distributions of H_Gut_Meta01 and H_Gut_Meta02 were more concentrated than those of H_Gut_Meta03 and P_Gut_Meta01 (Figure 3A). More than half of the assemblies with confident species‐level taxonomy had a genome fraction higher than 50%. Considering the large genetic diversity between sample genomes and reference genomes [8], these results suggested that our pipeline was able to obtain complete genomic information for the species with an abundance higher than 10×. The genetic diversity was also confirmed by the significant differences in genome fraction and the ratio of assembled length to the reference length among the four samples (Supporting Information: Figure S7). The distribution of genome N50 values was generally dispersed ranging from a few kb to several Mb, and the medians of H_Gut_Meta02 and H_Gut_Meta03 were higher than those of H_Gut_Meta01 and P_Gut_Meta01 (Figure 3B). Nevertheless, the third quartiles in the box plots for all the samples were larger than 100 kb, demonstrating that our pipeline has a strong capability to generate draft genomes with long contiguity. Note that from these three healthy samples, plenty of draft genomes with ultra‐long contiguity (N50 > 1 Mb) were obtained, which provide possibilities to study the large genome difference in the microbiome.

Considering the intra‐species genetic diversity, we also evaluated the quality of metagenomic assemblies by CheckM. The medians of the completeness of all assembled genomes in the three healthy samples were larger than 92%, while the medians of contamination were smaller than 2% (Figure 3C). The median completeness of the patient sample was about 83%, and the median contamination was about 7% (Supporting Information: Figure S8). A great number of high‐ and medium‐quality genomes were recovered by MetaTrass from the four samples (Figure 3D). Fifty‐two high‐quality and 37 medium‐quality genomes were produced for H_Gut_Meta01, 55 and 24 for H_Gut_Meta02, 47 and 16 for H_Gut_Meta03, and 24 and 28 for P_Gut_Meta01, respectively.

### Comparison to the assembly‐first‐and‐binning‐later strategies

To further evaluate our approach's efficiency, we compared it with conventional assembly‐first‐and‐binning‐later approaches as listed in the Section “Methods.” It should be noted that currently, there are still no genome binning tools to directly exploit the cobarcoding information. With regard to the number of genomes with a completeness of >50% (Supporting Information: Table S6), MetaTrass outperformed all the other methods on all data sets. Especially for P_Gut_Meta01, MetaTrass yielded 117 draft genomes with completeness higher than 50%, much more than the 66 ones obtained by the optimal combination of Supernova [39] and Maxbin2.0 [22].

**Figure 4:**
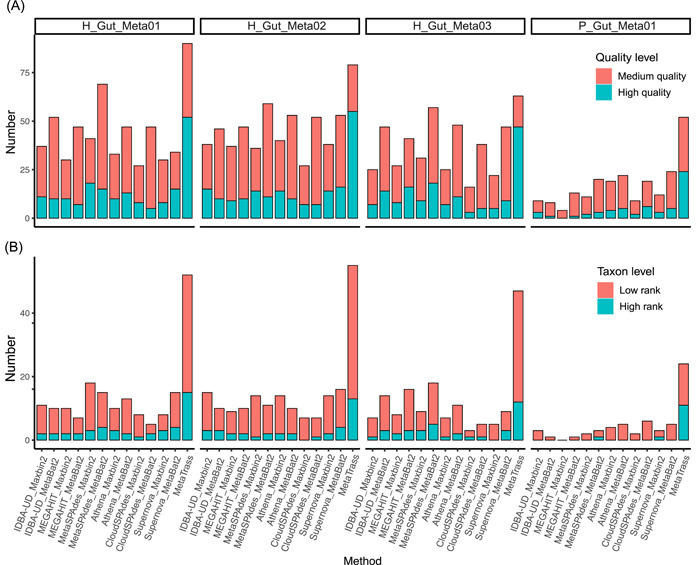
Comparison of metagenome assembly for different methods. (A) Number of high‐ and medium‐quality genomes assembled with different methods. (B) Number of high‐quality genomes with high‐ and low‐rank with different methods.

Through the comprehensive analysis of the completeness, contamination, and taxonomic rank of each genome, we assessed MetaTrass and conventional strategies on the ability to get high‐ and medium‐quality genomes and resolution of taxonomic rank (Figure 4). For different samples, the best combinations to produce optimal results were different. The combinations of MetaSPAdes [19] and Maxbin2.0, Supernova and MetaBAT2 [23], MetaSPAdes and MetaBAT2, and Athena [31] and MetaBAT2 were optimal for H_Gut_Meta01, H_Gut_Meta02, H_Gut_Meta03, and P_Gut_Meta01, respectively. For the four samples, the optimal results of the conventional strategies were still inferior to those of MetaTrass. For the example of H_Gut_Meta01, the combination of MetaSPAdes and Maxbin2.0 produced 41 high‐ and medium‐quality genomes, which was much less than the 90 obtained by MetaTrass. There were only 3 out of a total of 18 high‐quality genomes with a taxonomic rank lower than the bacterial order, but 15 out of 52 for MetaTrass. Compared with the strategies those only used NGS‐read information, MetaTrass outcompeted all these approaches by producing higher quality and finer resolution. These results demonstrated that the usage of cobarcoding information in MetaTrass was more efficient and accurate than those in the conventional approaches.

**Figure 5:**
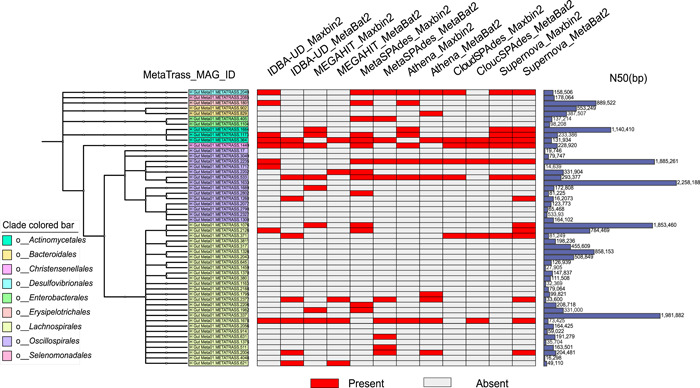
Taxonomic tree of the high‐quality genomes assembled by MetaTrass for H_Gut_Meta01. The taxonomic tree is on the left. The distribution of the high‐quality genomes assembled by other methods is colored red in the middle heatmap. N50 of each high‐quality genome is shown in the right histogram.

The human gut microbiome composition attracts much attention for many reasons, one of them being its strong correlation with personality traits [50]. To compare the microbiome composition of high‐quality genomes obtained with different methods, we uniformly used GTDB‐Tk [51] to annotate these genomes. The annotated taxonomic information was listed in Supporting Information: Table S7. Taxonomic trees of the high‐quality genomes obtained by MetaTrass were constructed based on the taxonomic information, and the corresponding N50 values were attached in the left histogram as shown in Figure 5. The high‐quality genomes obtained by the conventional strategies were marked in red in the middle of the heatmap (Figure 5) if the genome of the same species was also assembled by MetaTrass. The topology of the taxonomic tree of genomes assembled by MetaTrass gave comprehensive insights into the microbial composition structure. From the trees in Figure 5 and Supporting Information: Figures S9–S11, the numbers of the bacterial order with high‐quality genomes assembled by MetaTrass were 9, 11, 7, and 7 for H_Gut_Meta01, H_Gut_Meta02, H_Gut_Meta03, and P_Gut_Meta01, respectively. Notably, some bacterial orders contained more than five high‐quality genomes, and this could be used to study the microbiome structure at the genome‐wide scale in the same sample. For the sample H_Gut_Meta01 (Figure 5), there were 27 and 14 high‐quality genomes classified into the bacterial order *Lachnospirales* and *Oscillospirales*, respectively. These two were exactly the dominating bacterial orders according to the taxonomic abundance distribution. Similar results were obtained for the other two healthy samples (Supporting Information: Figures S9 and S10), indicating that the microbiome with higher sequencing coverage could be better assembled in MetaTrass. In contrast, the bacterial orders with more than five high‐quality genomes were *Enterobacterales* and *Actinomycetales* for P_Gut_Meta01 (Supporting Information: Figure S11). The obvious difference between the healthy and patient samples was consistent with the microbial composition differences observed in the taxonomic binning results. MetaTrass successfully assembled most of the high‐quality genomes of all the conventional strategies in our tests. Only 25 out of 137 genomes with high‐quality generated by all the conventional strategies were not assembled by MetaTrass (Figure 5). The heatmaps showed that most of the conventional strategies could assemble draft genomes for each bacterial order, but the numbers of genome in the same bacterial order were relatively small. The maximal number of genomes in one bacterial order was six and obtained by the combination of Supernova and MetaBAT2 for the bacterial order *Lachnospirales*. Moreover, 146 of 179 high‐quality genomes had N50 values larger than 100 kb, demonstrating that MetaTrass had a strong ability to improve the contiguity of assemblies.

### Genetic diversity in different samples

Different types of genomic variations in gut microbiomes are strongly associated with host health, and the genetic diversity among different microbiomes has been intensively studied to unravel the genetic origin of phenotypic differences among people of different geographical origins or health statuses [52, 53]. By aligning draft genomes to the references, we called variations for high‐quality genomes for each species in different samples, including single nucleotide variations (SNV) as well as small and large indels. For different variations, the number of SNVs was significantly larger than those of the small and large indels for the four samples (Supporting Information: Figure S12). Meanwhile, median values of variation numbers in the three healthy samples were similar but obviously larger than those in the patient sample. The smaller number of variations in the patient sample was due to the fewer alignments than those in the healthy samples according to the QUAST [54] evaluation. However, when we removed the effect of the total aligned length by calculating the SNV density, the patient sample showed denser SNV than the healthy samples (Supporting Information: Figure S12). The median was about 21 for the patient sample, but around nine for the healthy samples. This difference could be related to the individual's physiological state, which was related to the diseases, territory, and race [4].

**Figure 6:**
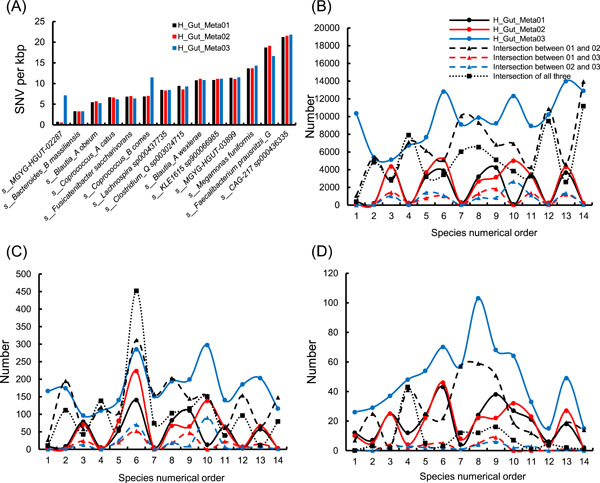
Single nucleotide variation (SNV) density and the number of unique and shared variations for each species appearing in all three healthy samples. (A) is the SNV density. (B), (C), and (D) are the number of SNVs, small, and large indels, respectively. The species numerical order in subfigures (B), (C), and (D) corresponds to the appearance order of species from left to right in subfigure A.

Based on the taxonomic information of the high‐quality genomes, we found 15 species shared by three samples, among which 14 species appeared in the three healthy samples and one species *Escherichia* appeared in the patient and two healthy samples. By analyzing the SNV density and intersection of variations between different samples for each species in three healthy samples, we further investigated the genetic diversity between species from different samples. The SNV densities were different for distinct species even in the same sample but were similar for the same species in different samples (Figure 6A). From Figure 6B–D, the number of unique and shared variations in different types significantly fluctuated for different species, but their difference among samples showed great consistency. H_Gut_Meta01 and H_Gut_Meta02 shared the highest number of variations among all sample pairs. Furthermore, the ratio of large indels shared by all three healthy samples to the total was much smaller than those of SNVs and small indels. These results demonstrated that large variations were more sample‐specific than small variations, which is consistent with previous observations of the association between host health and structural variations in the human gut microbiome [52].

### Computational performance

Runtime and used thread number of each assembler were recorded for all the human fecal data sets (Table 1). Most of the assemblers were tested on a small server of 24 Intel(R) Xeon(R) Silver 4116 CPU @ 2.10 GHz, except for Athena [31] and Supernova [39], which were tested on other high‐performance computing clusters due to their large memory requirements. The thread number set in each assembler was the same for different samples. The time consumption of the format conversion from stLFR reads to 10X linked‐reads was not included, which was about 500 min for a data set with 50 Gb in a single thread. We found that MetaTrass was less time‐consuming than Athena but more time‐consuming than other assemblers. This may be because both MetaTrass and Athena contained many subassembling, which took most of the time among all subprocesses in MetaTrass (Supporting Information: Table S8). Since the subassembling was independent, it could be run in parallel to further speed up by increasing the parallel number. Under the default parallel number of 8, the memory peaks used by MetaTrass were more than 50 Gb, but not more than 100 Gb for the four human fecal samples (Supporting Information: Table S9).

**Table 1:** Runtimes and thread number of each assembler for all the human gut data sets

	Thread number	Runtime (min)			
Assembler	All samples	H_Gut_meta01	H_Gut_Meta02	H_Gut_Meta03	P_Gut_Meta01
IBDA‐UD	6	863	884	911	2657
MEGAHIT	16	179	161	163	611
MetaSPAdes	16	1478	1289	1429	3459
CloudSPAdes	16	1024	1163	1039	2627
Supernova	8	1249	864	1098	6776
Athena	16	13,813	8689	6361	–
MetaTrass	16	5145	2631	3147	8363

*Note*: The exact runtime of assembling sample P_Gut_Meta01 by Athena was not collected correctly due to several uncontrolled interrupts on the high‐performance computing cluster.

## DISCUSSION

High‐quality genomes at the species level are strongly demanded to investigate the genetic origins of diseases associated with the human gut microbiome. However, how to get a sufficient number of them in one sample is still a challenge due to the inter‐species repeats and uneven abundance in metagenome assembly. In this study, we developed a tool to get high‐quality genomes with fine taxonomic resolutions by combining the cobarcoding information with public references. Compared with conventional strategies, our pipeline generated a large number of high‐quality genomes for the human microbiome cobarcoding data sets. Meanwhile, plenty of draft genomes were also assembled with an NG50 value larger than 1 Mb, some of which were even longer than the references for both the mock and human fecal data sets. For all four human fecal samples, 178 draft genomes with high completeness and low contamination were generated by MetaTrass, but their genome fractions relative to the references were low. The differences between the sample genomes assembled by MetaTrass and the reference genomes demonstrated that the cobarcoding information could be used to reduce the false‐negative reads in taxonomic binning. These reads retrieved from inter‐species repeat and unique genome‐specific regions by cobarcoded read refinement could significantly improve the quality of assemblies. For the patient sample, the number of high‐quality genomes with long contiguity assembled by MetaTrass was significantly larger than that generated without cobarcoded read refinement (Supporting Information: Figure S13).

MetaTrass was originally designed for stLFR, but is also suitable for other kinds of cobarcoding sequencing reads. We analyzed the 10× Genomics linked‐reads data set of ATCC Mock‐20 used in the development of Athena [31], which contains two bacterial genera with multiple species. As a result, MetaTrass recovered all these species with high‐quality genomes, while the conventional strategies only gained several metagenomes with various degrees of missing, heterogeneity, and contamination (Supporting Information: Figure S14). This also demonstrated the capability of MetaTrass to recover metagenomes at species or strain resolution. Noted that Supernova [39] was replaced with CloudSPAdes [42] in the step of cobarcoded read assembly. Since the number of long fragments with the same barcode in linked‐reads is greater than that of stLFR reads [32], more false‐positive reads were introduced into the cobarcoded refined read sets, leading to the unsuccessful assembling of several species by Supernova.

The efficiency of our pipeline depends on the cobarcoding information quality and the cobarcoding assembler. The cobarcoding information quality has strong effects on the precision and sensitivity of cobarcoded read refinement. By comparing genome fractions of different read sets including the TRs defined in the Section “Methods,” the refined reads, and all reads for species with medium abundance in P_Gut_Meta01 (Supporting Information: Table S10), we evaluated the sensitivity of the cobarcoded read refinement. We observed that the fraction with high depths of the refined reads was higher than that of the TRs, but still lower than that of all aligned reads. These results indicated that there were still some false‐negative reads introduced by the low coverage or the short length of long fragments. The cobarcoded read assembly of Supernova consumed most of the computational time in the tests of the four human fecal samples and it cannot effectively assemble data sets with a high ratio of false‐positive reads in the test of linked‐reads. Thus, improvements on the cobarcoding library and algorithms of the cobarcoded read refinement and assembly would improve the performance of MetaTrass.

## CONCLUSION

In summary, the application of MetaTrass in human fecal samples showed great promise in generating high‐quality genomes for a real complex microbial community at a fine resolution. With the increasing number of reference genomes from various microbial communities and the development of cobarcoding sequencing libraries, the binning‐first‐and‐assembly‐later strategy in MetaTrass will be strengthened and facilitate the investigation of the association between host phenotypes and microbial genotypes for different microbial communities.

## METHODS

### Data sets

A mock microbial and four human gut microbial communities were analyzed to evaluate the efficiency of MetaTrass. The mock microbial community (ZymoBIOMICS™ Microbial Community DNA Standard. Catalog D6305, Lot ZRC190812) consists of eight isolated bacteria and two fungi with an average abundance of about 12% and 2%, respectively (Supporting Information: Table S4). The four human gut microbial communities were sampled from the feces of three healthy volunteers and one patient with inflammatory bowel disease. The stLFR libraries were constructed according to the standard protocol [32]. The DNA samples were first sheared into long fragments, and then the long fragments were captured by magnetic microbeads with a unique barcode sequence. Finally, each long fragment was further fragmented and hybridized with a unique barcode by the Tn5 transposase on the surface of the microbead. The stLFR libraries of the mock microbial community and the patient sample were sequenced on the BGISEQ‐500 platform, and those of healthy samples were sequenced on the MGISEQ‐2000 platform. The length of paired‐end reads is 100 bp for all data sets. The three healthy sample libraries were individually allocated to a half lane and generated a total of about 50 Gb raw data on average. The mock and patient library was individually allocated to a full lane, resulting in about 85 and 100 Gb raw data, respectively. Barcode sequences were extracted from the end of the reverse read in a pair and then replaced with numerical symbols in the read names in the FASTQ file with our public tool stLFR_barcode_split [55]. SOAPfilter_v2.2 with parameters (‐y ‐F CTGTCTCTTATACACATCTTAGGAAGACAAGCACTGACGACATGA ‐R TCTGCTGAGTCGAGAACGTCTCTGTGAGCCAAGGAGTTGCTCTGG ‐p ‐M 2 ‐f ‐1 ‐Q 10) was used to remove low‐quality raw reads with adaptors, excessively confusing bases, and high duplications. Finally, 59.45 Gb of clean data were retained for the mock microbiome, 34.48 Gb for the first healthy sample (H_Gut_Meta01), 35.33 Gb for the second (H_Gut_Meta02), 37.88 Gb for the third (H_Gut_Meta03), and 97.20 Gb for the patient sample (P_Gut_Meta01). The clean data of all samples are available in the CNGB Sequence Archive (CNSA) [56] (https://db.cngb.org/cnsa/) of the China National GeneBank DataBase (CNGBdb) [57] with accession number CNP0002163.

### Taxonomic binning

We adopted Kraken2 (version 2.0.9‐beta) [44] to classify stLFR reads into different species. First, customized Kraken databases were constructed using the reference genomes of a studied microbial community. Then, the stLFR reads were classified according to the database. Both processes of Kraken were run with default parameters. Specifically, references attached to the ZYMO product were used to construct the database for the mock sample. The Kraken2 database of the UHGG collection was downloaded for the human fecal samples, which included 4542 representative genomes at the species level [4].

### Cobarcoded read refinement

A taxonomic tree of references was established to reduce the number of multiple hits of a *K‐mer* from inter‐species repeat sequences in Kraken2 [44]. The reads from these repetitive regions were classified into the lowest common ancient (LCA) rank higher than its corresponding species. Several previous studies tried to reallocate these reads to species by statistical inferences using the coverage depth or cobarcoding information [58, 59]. In the MetaTrass pipeline, the cobarcoding correlation between reads classified into a species and those classified into the LCA ranks higher than that species was used to reduce the false‐negative reads assigned to higher taxonomic ranks. Reads with a confident taxonomic assignment at the species level were treated as taxonomy reads (TRs) of the corresponding species. We collected and refined reads for each barcode according to the number of reads categorized as TRs (*Num_T*) and the ratio of these reads to the total reads (*Ratio_T*). Barcodes attached to TRs were first extracted as candidates. We ranked candidates according to *Num_T* in descending order first and then by *Ratio_T* also decreasingly. Finally, reads not more than 300× were collected to improve computational efficiency based on the rank of the barcodes.  Since sufficient read coverage is required for assembling a complete genome, only the species with an abundance higher than 10× were retained in this step. Reads assigned to these species were further clustered and filtered with the cobarcoding correlation. The abundance of each species was estimated by the quotient of the total base number of TRs to that of the reference. The refined reads of each species were extracted using Seqtk (version 1.3‐r114‐dirty) [60]. Note that there were still some false‐positive reads, probably resulting from the collision of long fragments from different species captured by the same microbead, even though *Ratio_T* was set to minimize the number. Sequences assembled from these reads would be further filtered out in the way described in the Section,  “Contamination removal.”

### Cobarcoded read assembly

Reads of a single species with an abundance higher than 10× were assembled in Supernova (version 2.1.1) [39], a high‐performance cobarcoding de novo assembler for single large eukaryotic genomes. Supernova was designed for linked‐reads of 10× Genomics (http://www.10xgenomics.com/), which have different barcodes and formats from stLFR reads. Thus, we converted the stLFR reads into linked‐reads FASTQ format before running Supernova. Additionally, the parameter—*accept‐extreme‐coverage* was set to *yes* to allow for large coverage depth differences.

### Contamination removal

As long fragments from different species could be captured by the same microbead in the stLFR library at low odds, reads from different species could putatively be labeled with the same barcode. As a result, a draft assembly of reads after cobarcoded read refinement might contain contaminated sequences from other species. We cleaned the assembly based on its similarity to the corresponding reference genome. The AF and ANI have been commonly adopted to circumscribe species [3, 4]. MetaTrass also adopted the metrics of AF and ANI. The ANI was independently calculated for each alignment. The AF was defined as the ratio of total alignment length with sufficiently high ANI to the total contig length. The alignments with ANI values higher than 90% were used in the AF calculation, and the contigs with AF values higher than 50% were retained to form the final assembly. The initial alignments between contigs and references were generated by QUAST (version 5.0.2) [54] with default parameters, except that the identity threshold to obtain valid alignment was set to 90%.

### Assembly‐first‐and‐binning‐later approaches

Conventional standard analysis pipelines of the NGS metagenomes implement de novo metagenome assembly first and further bin the contigs into draft genomes. We compared different existing assembly‐first‐and‐binning‐later approaches to MetaTrass by analyzing a mock and four human gut microbial communities. In our tests, the stLFR cobarcoded reads were assembled by NGS assemblers, including IDBA‐UD (version 1.1.3) [20], MEGAHIT (version 1.1.3) [18], and MetaSPAdes (version 3.10.1) [19], or cobarcoding assemblers, including Supernova [39], Athena (version 1.3.0) [31], and CloudSPAdes (version 3.13.1) [42]. Then, all the draft assemblies were binned by two genome binners, namely, MetaBAT2 (version 2.12.1) [23] and Maxbin2.0 (version 2.2.5) [22]. Since Supernova, CloudSPAdes, and Athena were not designed for stLFR reads, we made an appropriate format conversion with the step_2_10X_fake.sh script in our public tool stlfr2supernova_pipeline [61]. Except for Supernova, all the assemblers were run with default parameters. To make Supernova able to assemble data sets with extremely high coverage depths, the parameter—*accept‐extreme‐coverage* was set to *yes*. All assembly results are available in the CNSA [56] (https://db.cngb.org/cnsa/) of the CNGBdb [57] with accession number CNP0002163. MetaBAT2 and Maxbin2.0 were implemented with default parameters.

### Evaluations

Both reference‐based and reference‐free assessments were used to evaluate the quality of assemblies obtained using different strategies. For the mock microbial community, the reference‐based tool QUAST [54] was used to evaluate the contiguity and accuracy of metagenomic assemblies. Minimap2 (2.17‐r974‐dirty) [62] was used to map assemblies to references and get valid alignments with the identity threshold of 95%. Then, statistics such as genome fraction, NG50/NGA50, and the number of misassemblies were assessed from the alignments with default parameters. For the human gut microbial communities, the reference‐free tool CheckM (version 1.1.2) [63] was implemented with default parameters to evaluate the completeness and contamination of each genome. A definite number of marker genes conserved across almost all bacteria were used as the basis in CheckM. Following the guidance proposed in CheckM, we defined high‐quality assemblies (completeness > 90% and contamination < 5%) and medium‐quality assemblies (completeness > 50% and contamination < 10%, but not meet both completeness > 90% and contamination < 5%). In addition, the statistics of each genome notably N50, genome size, and taxonomic rank were also obtained by CheckM, and the taxonomic rank was used to show the resolution of a genome.

### Genomic variation estimation and taxonomic classification

All the high‐quality genomes assembled by MetaTrass were used to call variations for the four human fecal samples. We aligned each genome to their corresponding reference using Minimap2 [62] with parameters (*‐x asm5*) to prevent an alignment extending to regions with diversity >5%. SAMtools (version 1.9) [64] and PAFtools (2.17‐r982‐dirty) [65] were used to convert the BAM file of initial unsorted alignments into a PAF file of sorted alignments. We identified variations using the “call” module in PAFtools with parameters (*‐L 10000*) to filter out the alignments shorter than 10,000 bp. We considered single‐nucleotide substitutions as SNVs but ignored single‐base insertions or deletions. Insertions or deletions with lengths shorter than 50 bp were defined as small indels, and the rest were defined as large indels. Those genomic variations with the same position and sequence information that belong to the same species in different samples were determined as shared variations.

We used the “classify_wf” function of GTDB‐Tk (version 0.3.1) [51] to conduct taxonomic annotation of the genomes obtained by both MetaTrass and the conventional strategies with default parameters. Considering the procedure of the UHGG database construction [4], genomes were assigned at the species level if the AF to the close species representative genomes was higher than 30% and ANI was higher than 95%. Interactive Tree of Life (iTOL version 4.4.2) [66] was used to visualize taxonomic trees constructed from the annotated species information.

## AUTHOR CONTRIBUTIONS

Li Deng, Guangyi Fan, and Yanwei Qi contributed to the software design. Yanwei Qi, Shengqiang Gu, Yue Zhang, and Lidong Guo contributed to the software implementation. Li Deng, Yanwei Qi, Shengqiang Gu, Mengyang Xu, and Jianwei Chen contributed to the data analyses. Xiaofang Chen, Ou Wang, and Xiaodong Fang contributed to the data curation and collection. Guangyi Fan, Li Deng, and Xin Liu contributed to the benchmarking design. All authors contributed to the manuscript writing. Li Deng, Yanwei Qi, Lidong Guo, Mengyang Xu, and Ying Sun contributed to the manuscript revision. Li Deng and Guangyi Fan supervised the project. All authors read and approved the final manuscript.

## CONFLICT OF INTEREST

The authors declare no conflict of interest.

## Supporting information

Supplementary information.

Supplementary information.

## Data Availability

MetaTrass is a command‐line application released to GitHub as open‐source software under General Public License v3.0 (https://github.com/BGI-Qingdao/MetaTrass). The assembling results and cleaned stLFR data sets of the mock and four human fecal samples were deposited into CNSA (https://db.cngb.org/cnsa/) of CNGBdb with accession number CNP0002163 and available from authors upon reasonable application through CNGBdb. The 10X Genomics linked‐reads data sets of Mock‐20 were downloaded from the website https://trace.ncbi.nlm.nih.gov/Traces/index.html?run=SRR6760785. Supplementary materials may be found in the online DOI or iMeta Science http://www.imeta.science/

## References

[1] Schloss, Patrick D. , and Jo Handelsman . 2005. “Metagenomics for Studying Unculturable Microorganisms: Cutting the Gordian Knot.” Genome Biology 6: 1–4. 10.1186/gb-2005-6-8-229 PMC1273625 16086859

[2] Qin, Junjie , Ruiqiang Li , Jeroen Raes , Manimozhiyan Arumugam , Kristoffer Solvsten Burgdorf , Chaysavanh Manichanh , Trine Nielsen , et al. 2010. “A Human Gut Microbial Gene Catalogue Established by Metagenomic Sequencing.” Nature 464: 59–65. 10.1038/nature08821 20203603 PMC3779803

[3] Parks, Donovan H. , Maria Chuvochina , Pierre‐Alain Chaumeil , Christian Rinke , Aaron J. Mussig , and Philip Hugenholtz . 2020. “A Complete Domain‐to‐Species Taxonomy for Bacteria and Archaea.” Nature Biotechnology 38: 1079–86. 10.1038/s41587-020-0501-8 32341564

[4] Almeida, Alexandre , Stephen Nayfach , Miguel Boland , Francesco Strozzi , Martin Beracochea , Zhou Jason Shi , Katherine S. Pollard , et al. 2021. “A Unified Catalog of 204,938 Reference Genomes from the Human Gut Microbiome.” Nature Biotechnology 39: 105–14. 10.1038/s41587-020-0603-3 PMC7801254 32690973

[5] Sheth, Ravi U. , Mingqiang Li , Weiqian Jiang , Peter A. Sims , Kam W. Leong , and Harris H. Wang . 2019. “Spatial Metagenomic Characterization of Microbial Biogeography in the Gut.” Nature Biotechnology 37: 877–83. 10.1038/s41587-019-0183-2 PMC6679743 31332325

[6] Martino, Cameron , Liat Shenhav , Clarisse A. Marotz , George Armstrong , Daniel McDonald , Yoshiki Vázquez‐Baeza , James T. Morton , et al. 2021. “Context‐Aware Dimensionality Reduction Deconvolutes Gut Microbial Community Dynamics.” Nature Biotechnology 39: 165–8. 10.1038/s41587-020-0660-7 PMC7878194 32868914

[7] Qian, Xubo , Tong Chen , Yiping Xu , Lei Chen , Fuxiang Sun , Meiping Lu , and Yongxin Liu . 2020. “A Guide to Human Microbiome Research: Study Design, Sample Collection, and Bioinformatics Analysis.” Chinese Medical Journal 133: 1844–55. 10.1097/cm9.0000000000000871 32604176 PMC7469990

[8] Van Rossum, Thea , Pamela Ferretti , Oleksandr M. Maistrenko , and Peer Bork . 2020. “Diversity within Species: Interpreting Strains in Microbiomes.” Nature Reviews: Microbiology 18: 491–506. 10.1038/s41579-020-0368-1 32499497 PMC7610499

[9] Olm, Matthew R. , Alexander Crits‐Christoph , Keith Bouma‐Gregson , Brian A. Firek , Michael J. Morowitz , and Jillian F. Banfield . 2021. “Instrain Profiles Population Microdiversity from Metagenomic Data and Sensitively Detects Shared Microbial Strains.” Nature Biotechnology 39: 727–36. 10.1038/s41587-020-00797-0 PMC9223867 33462508

[10] Leimbach, Andreas , Jörg Hacker , and Ulrich Dobrindt . 2013. “ *E. Coli* as an All‐Rounder: The Thin Line between Commensalism and Pathogenicity.” Current Topics in Microbiology and Immunology 358: 3–32. 10.1007/82_2012_303 23340801

[11] Pierce, Jessica V. , and Harris D. Bernstein . 2016. “Genomic Diversity of Enterotoxigenic Strains of *Bacteroides Fragilis* .” PLoS One 11: e0158171. 10.1371/journal.pone.0158171 27348220 PMC4922554

[12] Yao, Guocai , Wenliang Zhang , Minglei Yang , Huan Yang , Jianbo Wang , Haiyue Zhang , Lai Wei , Zhi Xie , and Weizhong Li . 2020. “MicroPhenoDB Associates Metagenomic Data with Pathogenic Microbes, Microbial Core Genes, and Human Disease Phenotypes.” Genomics Proteomics Bioinformatics 18: 760–72. 10.1016/j.gpb.2020.11.001 33418085 PMC8377004

[13] Welch, R. A. , Valerie Burland , Guy Plunkett , Peter Redford , Paula Roesch , David A. Rasko , Eric. L. Buckles , et al. 2002. “Extensive Mosaic Structure Revealed by the Complete Genome Sequence of Uropathogenic *Escherichia Coli* .” Proceedings of the National Academy of Sciences of the United States of America 99: 17020–4. 10.1073/pnas.252529799 12471157 PMC139262

[14] Liu, Yongxin , Yuan Qin , Tong Chen , Meiping Lu , Xubo Qian , Xiaoxuan Guo , and Yang Bai . 2021. “A Practical Guide to Amplicon and Metagenomic Analysis of Microbiome Data.” Protein & Cell 12: 315–30. 10.1007/s13238-020-00724-8 32394199 PMC8106563

[15] Sczyrba, Alexander , Peter Hofmann , Peter Belmann , David Koslicki , Stefan Janssen , Johannes Dröge , Ivan Gregor , et al. 2017. “Critical Assessment of Metagenome Interpretation—A Benchmark of Metagenomics Software.” Nature Methods 14: 1063–71. 10.1038/nmeth.4458 28967888 PMC5903868

[16] Breitwieser, Florian P. , Jennifer Lu , and Steven L. Salzberg . 2019. “A Review of Methods and Databases for Metagenomic Classification and Assembly.” Briefings in Bioinformatics 20: 1125–36. 10.1093/bib/bbx120 29028872 PMC6781581

[17] Eun Kang, Jee , Antonio Ciampi , and Mohamed Hijri . 2020. “SeSaMe: Metagenome Sequence Classification of Arbuscular Mycorrhizal Fungi‐Associated Microorganisms.” Genomics Proteomics Bioinformatics 18: 601–12. 10.1016/j.gpb.2018.07.010 33346086 PMC8377386

[18] Li, Dinghua , Chi‐Man Liu , Ruibang Luo , Kunihiko Sadakane , and Tak‐Wah Lam . 2015. “MEGAHIT: An Ultra‐Fast Single‐Node Solution for Large and Complex Metagenomics Assembly Via Succinct De Bruijn Graph.” Bioinformatics 31: 1674–6. 10.1093/bioinformatics/btv033 25609793

[19] Nurk, Sergey , Dmitry Meleshko , Anton Korobeynikov , and Pavel A. Pevzner . 2017. “metaSPAdes: A New Versatile Metagenomic Assembler.” Genome Research 27: 824–34. 10.1101/gr.213959.116 28298430 PMC5411777

[20] Peng, Yu , Henry C. M. Leung , Siu‐Ming Yiu , and Francis Y. L. Chin . 2012. “IDBA‐UD: A *de novo* Assembler for Single‐Cell and Metagenomic Sequencing Data with Highly Uneven Depth.” Bioinformatics 28: 1420–8. 10.1093/bioinformatics/bts174 22495754

[21] Wu, Yu‐Wei , and Yuzhen Ye . 2011. “A Novel Abundance‐Based Algorithm for Binning Metagenomic Sequences Using l‐Tuples.” Journal of Computational Biology 18: 523–34. 10.1089/cmb.2010.0245 21385052 PMC3123841

[22] Wu, Yu‐Wei , Blake A. Simmons , and Steven W. Singer . 2016. “MaxBin 2.0: An Automated Binning Algorithm to Recover Genomes from Multiple Metagenomic Datasets.” Bioinformatics 32: 605–7. 10.1093/bioinformatics/btv638 26515820

[23] Kang, Dongwan D. , Jeff Froula , Rob Egan , and Zhong Wang . 2015. “MetaBAT, an Efficient Tool for Accurately Reconstructing Single Genomes from Complex Microbial Communities.” PeerJ 3: e1165. 10.7717/peerj.1165 26336640 PMC4556158

[24] Bertrand, Denis , Jim Shaw , Manesh Kalathiyappan , Amanda Hui Qi Ng , M. Senthil Kumar , Chenhao Li , Mirta Dvornicic , et al. 2019. “Hybrid Metagenomic Assembly Enables High‐Resolution Analysis of Resistance Determinants and Mobile Elements in Human Microbiomes” Nature Biotechnology 37: 937–44. 10.1038/s41587-019-0191-2 31359005

[25] Chin, Chen‐Shan , David H. Alexander , Patrick Marks , Aaron A. Klammer , James Drake , Cheryl Heiner , Alicia Clum , et al. 2013. “Nonhybrid, Finished Microbial Genome Assemblies from Long‐Read SMRT Sequencing Data.” Nature Methods 10: 563–9. 10.1038/nmeth.2474 23644548

[26] Kolmogorov, Mikhail , Derek M. Bickhart , Bahar Behsaz , Alexey Gurevich , Mikhail Rayko , Sung Bong Shin , Kristen Kuhn , et al. 2020. “Metaflye: Scalable Long‐Read Metagenome Assembly Using Repeat Graphs.” Nature Methods 17: 1103–10. 10.1038/s41592-020-00971-x 33020656 PMC10699202

[27] DeMaere, Matthew Z. , and Aaron E. Darling . 2019. “bin3C: Exploiting Hi‐C Sequencing Data to Accurately Resolve Metagenome‐Assembled Genomes.” Genome Biology 20: 1–16. 10.1186/s13059-019-1643-1 30808380 PMC6391755

[28] Cleary, Brian , Ilana Lauren Brito , Katherine Huang , Dirk Gevers , Terrance Shea , Sarah Young , and Eric J. Alm . 2015. “Detection of Low‐Abundance Bacterial Strains in Metagenomic Datasets by Eigengenome Partitioning.” Nature Biotechnology 33: 1053–60. 10.1038/nbt.3329 PMC4720164 26368049

[29] Peters, Brock A. , Bahram G. Kermani , Andrew B. Sparks , Oleg Alferov , Peter Hong , Andrei Alexeev , Yuan Jiang , et al. 2012. “Accurate Whole‐Genome Sequencing and Haplotyping from 10 to 20 Human Cells.” Nature 487: 190–5. 10.1038/nature11236 22785314 PMC3397394

[30] Adey, Andrew , Jacob O. Kitzman , Joshua N. Burton , Riza Daza , Akash Kumar , Lena Christiansen , Mostafa Ronaghi , et al. 2014. “In Vitro, Long‐Range Sequence Information for *de novo* Genome Assembly Via Transposase Contiguity.” Genome Research 24: 2041–9. 10.1101/gr.178319.114 25327137 PMC4248320

[31] Bishara, Alex , Eli L. Moss , Mikhail Kolmogorov , Alma E. Parada , Ziming Weng , Arend Sidow , Anne E. Dekas , Serafim Batzoglou , and Ami S. Bhatt . 2018. “High‐Quality Genome Sequences of Uncultured Microbes by Assembly of Read Clouds” Nature Biotechnology 36: 1067–75. 10.1038/nbt.4266 PMC6465186 30320765

[32] Wang, Ou , Robert Chin , Xiaofang Cheng , Michelle Wu , Qing Mao , Jingbo Tang , Yuhui Sun , et al. 2019. “Efficient and Unique Cobarcoding of Second‐Generation Sequencing Reads from Long DNA Molecules Enabling Cost‐Effective and Accurate Sequencing, Haplotyping, and *de novo* Assembly.” Genome Research 29: 798–808. 10.1101/gr.245126.118 30940689 PMC6499310

[33] Chen, Zhoutao , Long Pham , Tsai‐Chin Wu , Guoya Mo , Yu Xia , Peter L. Chang , Devin Porter , et al. 2020. “Ultralow‐Input Single‐Tube Linked‐Read Library Method Enables Short‐Read Second‐Generation Sequencing Systems to Routinely Generate Highly Accurate and Economical Long‐Range Sequencing Information.” Genome Research 30: 898–909. 10.1101/gr.260380.119 32540955 PMC7370886

[34] Zheng, Grace X. Y. , Billy T. Lau , Michael Schnall‐Levin , Mirna Jarosz , John M. Bell , Christopher M. Hindson , Sofia Kyriazopoulou‐Panagiotopoulou , et al. 2016. “Haplotyping Germline and Cancer Genomes with High‐Throughput Linked‐Read Sequencing.” Nature Biotechnology 34: 303–11. 10.1038/nbt.3432 PMC4786454 26829319

[35] Danko, David C. , Dmitry Meleshko , Daniela Bezdan , Christopher Mason , and Iman Hajirasouliha . 2019. “Minerva: An Alignment‐and Reference‐Free Approach to Deconvolve Linked‐Reads for Metagenomics.” Genome Research 29: 116–24. 10.1101/gr.235499.118 30523036 PMC6314158

[36] Xu, Mengyang , Lidong Guo , Xiao Du , Lei Li , Brock A. Peters , Li Deng , Ou Wang , et al. 2021. “Accurate Haplotype‐Resolved Assembly Reveals the Origin of Structural Variants for Human Trios.” Bioinformatics 37: 2095–102. 10.1093/bioinformatics/btab068 33538292 PMC8613828

[37] Bishara, Alex , Yuling Liu , Ziming Weng , Dorna Kashef‐Haghighi , Daniel E. Newburger , Robert West , Arend Sidow , and Serafim Batzoglou . 2015. “Read Clouds Uncover Variation in Complex Regions of the Human Genome.” Genome Research 25: 1570–80. 10.1101/gr.191189.115 26286554 PMC4579342

[38] Guo, Junfu , Chang Shi , Xi Chen , Ou Wang , Ping Liu , Huanming Yang , Xun Xu , Wenwei Zhang , and Hongmei Zhu . 2021. “stLFRsv: A Germline Structural Variant Analysis Pipeline Using Co‐Barcoded Reads.” Frontiers in Genetics 12: 222. 10.3389/fgene.2021.636239 PMC8012683 33815469

[39] Weisenfeld, Neil I. , Vijay Kumar , Preyas Shah , Deanna M. Church , and David B. Jaffe . 2017. “Direct Determination of Diploid Genome Sequences.” Genome Research 27: 757–67. 10.1101/gr.214874.116 28381613 PMC5411770

[40] Yeo, Sarah , Lauren Coombe , René L Warren , Justin Chu , and Inanç Birol . 2017. “ARCS: Scaffolding Genome Drafts with Linked Reads.” Bioinformatics 34: 725–31. 10.1093/bioinformatics/btx675 PMC6030987 29069293

[41] Guo, Lidong , Mengyang Xu , Wenchao Wang , Shengqiang Gu , Xia Zhao , Fang Chen , Ou Wang , et al. 2021. “SLR‐superscaffolder: A *de novo* Scaffolding Tool for Synthetic Long Reads Using a Top‐to‐Bottom Scheme.” BMC Bioinformatics 22: 1–16. 10.1186/s12859-021-04081-z 33765921 PMC7993450

[42] Tolstoganov, Ivan , Anton Bankevich , Zhoutao Chen , and Pavel A. Pevzner . 2019. “cloudSPAdes: Assembly of Synthetic Long Reads Using De Bruijn Graphs.” Bioinformatics 35: i61–70. 10.1093/bioinformatics/btz349 31510642 PMC6612831

[43] Kuleshov, Volodymyr , Michael P. Snyder , and Serafim Batzoglou . 2016. “Genome Assembly From Synthetic Long Read Clouds.” Bioinformatics 32: i216–24. 10.1093/bioinformatics/btw267 27307620 PMC4908351

[44] Wood, Derrick E. , Jennifer Lu , and Ben Langmead . 2019. “Improved Metagenomic Analysis with Kraken 2.” Genome Biology 20: 1–13. 10.1186/s13059-019-1891-0 31779668 PMC6883579

[45] Koren, Sergey , Arang Rhie , Brian P. Walenz , Alexander T. Dilthey , Derek M. Bickhart , Sarah B. Kingan , Stefan Hiendleder , et al. 2018. “ *de novo* Assembly of Haplotype‐Resolved Genomes with Trio Binning.” Nature Biotechnology 36: 1174–82. 10.1038/nbt.4277 PMC6476705 30346939

[46] Nicholls, Samuel M. , Joshua C. Quick , Shuiquan Tang , and Nicholas J. Loman . 2019. “Ultra‐Deep, Long‐Read Nanopore Sequencing of Mock Microbial Community Standards.” GigaScience 8: giz043. 10.1093/gigascience/giz043 31089679 PMC6520541

[47] Ruan, Jue , and Heng Li . 2020. “Fast and Accurate Long‐Read Assembly with wtdbg2.” Nature Methods 17: 155–8. 10.1038/s41592-019-0669-3 31819265 PMC7004874

[48] Bankevich, Anton , Sergey Nurk , Dmitry Antipov , Alexey A. Gurevich , Mikhail Dvorkin , Alexander S. Kulikov , Valery M. Lesin , et al. 2012. “SPAdes: A New Genome Assembly Algorithm and its Applications to Single‐Cell Sequencing.” Journal of Computational Biology 19: 455–77. 10.1089/cmb.2012.0021 22506599 PMC3342519

[49] Shin, Na‐Ri , Tae Woong Whon , and Jin‐Woo Bae . 2015. “ *Proteobacteria*: Microbial Signature of Dysbiosis in Gut Microbiota.” Trends in Biotechnology 33: 496–503. 10.1016/j.tibtech.2015.06.011 26210164

[50] Johnson, Katerina V‐A. 2020. “Gut Microbiome Composition and Diversity Are Related to Human Personality Traits.” Human Microbiome Journal 15: 100069. 10.1016/j.humic.2019.100069 PMC8336012 34435164

[51] Chaumeil, Pierre‐Alain , Aaron J. Mussig , Philip Hugenholtz , and Donovan H. Parks . 2019. “GTDB‐Tk: A Toolkit to Classify Genomes with the Genome Taxonomy Database.” Bioinformatics 36: 1925–7. 10.1093/bioinformatics/btz848 31730192 PMC7703759

[52] Zeevi, David , Tal Korem , Anastasia Godneva , Noam Bar , Alexander Kurilshikov , Maya Lotan‐Pompan , Adina Weinberger , et al. 2019. “Structural Variation in the Gut Microbiome Associates with Host Health.” Nature 568: 43–8. 10.1038/s41586-019-1065-y 30918406

[53] Chen, Lianmin , Daoming Wang , Sanzhima Garmaeva , Alexander Kurilshikov , Arnau Vich Vila , Ranko Gacesa , Trishla Sinha , et al. 2021. “The Long‐Term Genetic Stability and Individual Specificity of the Human Gut Microbiome.” Cell 184: 2302–15. 10.1016/j.cell.2021.03.024 33838112

[54] Gurevich, Alexey , Vladislav Saveliev , Nikolay Vyahhi , and Glenn Tesler . 2013. “QUAST: Quality Assessment Tool for Genome Assemblies.” Bioinformatics 29: 1072–5. 10.1093/bioinformatics/btt086 23422339 PMC3624806

[55] Guo, Lidong . stLFR_barcode_split. https://github.com/BGI-Qingdao/stLFR_barcode_split.

[56] Guo, Xueqin , Fengzhen Chen , Fei Gao , Ling Li , Ke Liu , Lijin You , Cong Hua , et al. 2020. “CNSA: A Data Repository for Archiving Omics Data.” Database 2020: baaa055. 10.1093/database/baaa055. 32705130 PMC7377928

[57] Chen, Fengzhen , Lijin You , Fan Yang , Lina Wang , Xueqin Guo , Fei Gao , Cong Hua , et al. 2020. “CNGBdb: China National Genebank Database.” Heredidas 42: 799–809. 10.16288/j.yczz.20-080 32952115

[58] Lu, Jennifer , Florian P. Breitwieser , Peter Thielen , and Steven L. Salzberg . 2017. “Bracken: Estimating Species Abundance in Metagenomics Data.” PeerJ Computer Science 3: e104. 10.7717/peerj-cs.104 PMC12016282 40271438

[59] Danko, David C. , Dmitry Meleshko , Daniela Bezdan , Christopher Mason , and Iman Hajirasouliha . 2019. Novel Algorithms for the Taxonomic Classification of Metagenomic Linked‐Reads. *bioRxiv* 549667. 10.1101/549667

[60] Li, Heng . seqtk. https://github.com/lh3/seqtk

[61] Guo, Lidong . stlfr2supernova_pipeline. https://github.com/BGI-Qingdao/stlfr2supernova_pipeline

[62] Li, Heng . 2018. “Minimap2: Pairwise Alignment for Nucleotide Sequences.” Bioinformatics 34: 3094–3100. 10.1093/bioinformatics/bty191 29750242 PMC6137996

[63] Parks, Donovan H. , Michael Imelfort , Connor T. Skennerton , Philip Hugenholtz , and Gene W. Tyson . 2015. “CheckM: Assessing the Quality of Microbial Genomes Recovered from Isolates, Single Cells, and Metagenomes.” Genome Research 25: 1043–1055. 10.1101/gr.186072.114 25977477 PMC4484387

[64] Li, Heng , Bob Handsaker , Alec Wysoker , Tim Fennell , Jue Ruan , Nils Homer , Gabor Marth , et al. 2009. “The Sequence Alignment/Map Format and SAMtools.” Bioinformatics 25: 2078–9. 10.1093/bioinformatics/btp352 19505943 PMC2723002

[65] Li, Heng . paftools. https://github.com/lh3/minimap2/tree/master/misc

[66] Letunic, Ivica , and Peer Bork . 2019. “Interactive Tree of Life (iTOL) v4: Recent Updates and New Developments” Nucleic Acids Research 47: W256–9. 10.1093/nar/gkz239 30931475 PMC6602468

