# Correction to “MetaTrass: A high‐quality metagenome assembler of the human gut microbiome by cobarcoding sequencing reads”

**DOI:** 10.1002/imt2.150

**Published:** 2023-11-06

In Qi et al. [1], the following ethics statement should have been added.

The ethics application (No. BGI‐R052‐4 and BGI‐R052‐4‐T1) was approved by The Institutional Review Board of BGI.

The authors wish to apologize for the oversight.
