# Supplementary material for: MetaTrass: A high‐quality metagenome assembler of the human gut microbiome by cobarcoding sequencing reads
Source: Imeta. 2022 Aug 15;1(4):e46. doi: 10.1002/imt2.46 (PMC10989976; doi:10.1002/imt2.46)
Supplement: Supplementary file 1 — Supplementary information. [file IMT2-1-e46-s001.docx]

**SUPPORTING INFORMTION**

**MetaTrass: a high-quality metagenome assembler of the human gut microbiome by cobarcoding sequencing reads**

Yanwei Qi^1,2,3#^, Shengqiang Gu^1,4#^, Yue Zhang^1^, Lidong Guo^1,4^, Mengyang Xu^1,2,3,5^, Xiaofang Cheng^5,6^, Ou Wang^5,6^, Ying Sun^1^, Jianwei Chen^1^, Xiaodong Fang^5,7^, Xin Liu^1,2,3^, Li Deng^1,2,3*^, Guangyi Fan^1,2,3,5*^

^1^ BGI-Qingdao, BGI-Shenzhen, Qingdao 266555, China

^2^ State Key Laboratory of Agricultural Genomics, BGI-Shenzhen, Shenzhen 518083, China

^3^ China National GeneBank, BGI-Shenzhen, Shenzhen 518120, China

^4^ College of Life Sciences, University of Chinese Academy of Sciences, Beijing 100049, China

^5^ BGI-Shenzhen, Shenzhen 518083, China

^6^ MGI, BGI-Shenzhen, Shenzhen 518083, China

^7^ BGI Genomics, BGI-Shenzhen, Shenzhen 518083, China

**Supplementary figures**

**Figure S1** The probability of barcodes with long fragments from different species for four human faecal samples.

**Figure S2** Mismatches and indels of different assemblies for the mock dataset.

**Figure S3** The distribution of classified reads across different bacterial phyla for four human faecal samples.

**Figure S4** The distribution of classified reads across different bacterial classes for four human faecal samples.

**Figure S5** The distribution of classified reads across different bacterial orders for four human faecal samples.

**Figure S6** The distribution of classified reads across different bacterial families for four human faecal samples.

**Figure S7** Genome faction and the ratio of the assembly length to the reference length of all assembled species by MetaTrass for four human faecal samples. The genomes are sorted by the completeness evaluated by CheckM.

**Figure S8** Two-dimensional scatter plot of completeness and contamination evaluated by CheckM for four human faecal samples.

**Figure S9** Taxonomic tree of the high-quality genomes assembled by MetaTrass for H_Gut_Meta02. The taxonomic tree is on the left. The distribution of the high-quality genomes assembled by other methods is colored red in the middle heatmap. N50 of each high-quality genome is shown in the right histogram.

**Figure S10** Taxonomic tree of the high-quality genomes assembled by MetaTrass for H_Gut_Meta03. The taxonomic tree is on the left. The distribution of the high-quality genomes assembled by other methods is colored red in the middle heatmap. N50 of each high-quality genome is shown in the right histogram.

**Figure S11** Taxonomic tree of the high-quality genomes assembled by MetaTrass for P_Gut_Meta01**.** The taxonomic tree is on the left. N50 of each high-quality genome is shown in the right histogram. Because most of the genomes obtained by the combination strategies cannot be classified into species level by GTDB-Tk, the heatmap is not shown for this sample.

**Figure S12** Box plot of variations. Box plots of single nucleotide variants (SNVs), small indels, large indels, and SNV density called from the high-quality genomes for four faecal samples.

**Figure S13** Number of genomes with different quality (A) and contiguity (B) assembled by MetaTrass and MetaTrass_TR for the patient gut sample. Since MetaTrass_TR excluded the cobarcoded read refinement process compared to MetaTrass, the input dataset of cobarcoded read assembly in MetaTrass_TR is the taxonomy read set. The number of genomes with different contiguity levels in (B) was counted for the high-quality genomes in (A).

**Figure S14** Assembling results of linked-reads for the sample Mock-20. Taxonomic tree of the references and the heatmap of the distribution of genomes assembled by different methods (A). Evaluation results of draft genomes of the species belonging to the same genus by CheckM (B).
